# Antipsychotics-Induced Changes in Synaptic Architecture and Functional Connectivity: Translational Implications for Treatment Response and Resistance

**DOI:** 10.3390/biomedicines10123183

**Published:** 2022-12-08

**Authors:** Andrea de Bartolomeis, Giuseppe De Simone, Mariateresa Ciccarelli, Alessia Castiello, Benedetta Mazza, Licia Vellucci, Annarita Barone

**Affiliations:** Section of Psychiatry, Laboratory of Translational and Molecular Psychiatry and Unit of Treatment-Resistant Psychosis, Department of Neuroscience, Reproductive Sciences and Odontostomatology, University Medical School of Naples “Federico II”, Via Pansini 5, 80131 Naples, Italy

**Keywords:** brain network, dopamine, glutamate, serotonin, postsynaptic density, connectivity, schizophrenia, immediate early genes, antipsychotics, Homer

## Abstract

Schizophrenia is a severe mental illness characterized by alterations in processes that regulate both synaptic plasticity and functional connectivity between brain regions. Antipsychotics are the cornerstone of schizophrenia pharmacological treatment and, beyond occupying dopamine D2 receptors, can affect multiple molecular targets, pre- and postsynaptic sites, as well as intracellular effectors. Multiple lines of evidence point to the involvement of antipsychotics in sculpting synaptic architecture and remodeling the neuronal functional unit. Furthermore, there is an increasing awareness that antipsychotics with different receptor profiles could yield different interregional patterns of co-activation. In the present systematic review, we explored the fundamental changes that occur under antipsychotics’ administration, the molecular underpinning, and the consequences in both acute and chronic paradigms. In addition, we investigated the relationship between synaptic plasticity and functional connectivity and systematized evidence on different topographical patterns of activation induced by typical and atypical antipsychotics.

## 1. Introduction

Seventy years after their introduction in therapy, antipsychotics still represent the cornerstone of schizophrenia pharmacological treatment as well as a relevant therapy, along with mood stabilizers, in the treatment of bipolar disorders and a validated strategy for augmenting antidepressants’ action [1,2]. Most antipsychotics, albeit with individual differences, share the common feature of occupying the dopamine D2 receptor (D2R), which is considered the main mechanism responsible for their therapeutic effect [3]. Despite the relevance of dopamine-related action, atypical antipsychotics and novel compounds in experimental trials modulate other neurotransmitter pathways, including the serotonergic, glutamatergic, and adrenergic ones with a putative beneficial effect on psychotic symptoms [4,5,6]. Mounting evidence suggests that antipsychotics can lead to morphological and synaptic changes in the brain through the modulation of processes that regulate synaptic plasticity, dendritic spine architecture, and postsynaptic density [5,7]. In addition, antipsychotics may promote a functional reorganization of neural connections and modify the correlation pattern existing between pairs of brain regions [8,9].

The investigation of antipsychotics’ mechanism of action at a molecular and network level is particularly relevant for those diseases that are conceptualized as disorders of both synaptic plasticity and brain cortical-subcortical connectivity, such as schizophrenia [10,11,12]. Specifically, several studies have supported the neurodevelopmental hypothesis for schizophrenia by the direct detection of synaptic plasticity and postsynaptic density (PSD) alterations in patients with psychosis compared to controls [13,14]. The PSD, which is a major contributor to changes in synaptic architecture under multiple stimuli including antipsychotics’ administration, is a highly organized structure beneath the neuronal membrane mainly at glutamate synapses and comprises receptors, scaffolding, and adaptor proteins [15,16]. Moreover, in recent years, the psychopathological manifestations of schizophrenia have been attributed to the loss of neuronal synchrony ensuing in a global dysconnectivity between and within brain networks [17]. Thus, even though the anatomical alteration in schizophrenia is placed at a microscopic level, for instance at PSD, the resulting macroscale manifestation is the impairment in functional interactions among brain regions [18].

Here, we addressed the following questions:

1. How do changes in gene expression induced by antipsychotics impact brain synapses and connectivity?
2. Where do these changes occur in terms of brain region topography?
3. Are the brain regions involved coherent with the modeling of schizophrenia pathophysiology?
4. And finally, how could these changes translate in terms of implications for response and resistance to antipsychotic treatment?

To answer these questions, we described here the molecular mechanisms underlying the interrelationship between changes in synaptic plasticity, mainly PSD, and brain functional connectivity, assessing the impact of antipsychotics on synaptic architecture and neuronal activity, as well as highlighting differences in the topographical pattern of co-activation induced by different antipsychotics alone or in combination. Further, we analyzed the regional expression of immediate early genes (IEGs) after the administration of antipsychotics, which increases rapidly and transiently in response to neuronal activation and represents a valuable marker of postsynaptic enhancement [19]. In addition, we recapitulated the major findings from preclinical studies investigating the impact of molecules on brain functional connectivity and matched this information with putative beneficial antipsychotic effects. Lastly, we critically evaluated the methods used to explore connectivity in preclinical models of schizophrenia, focusing on the pros and cons of each procedure and pointing to novel future applications for responsiveness and resistance to antipsychotics.

## 2. Materials and Methods

**Figure 1:**
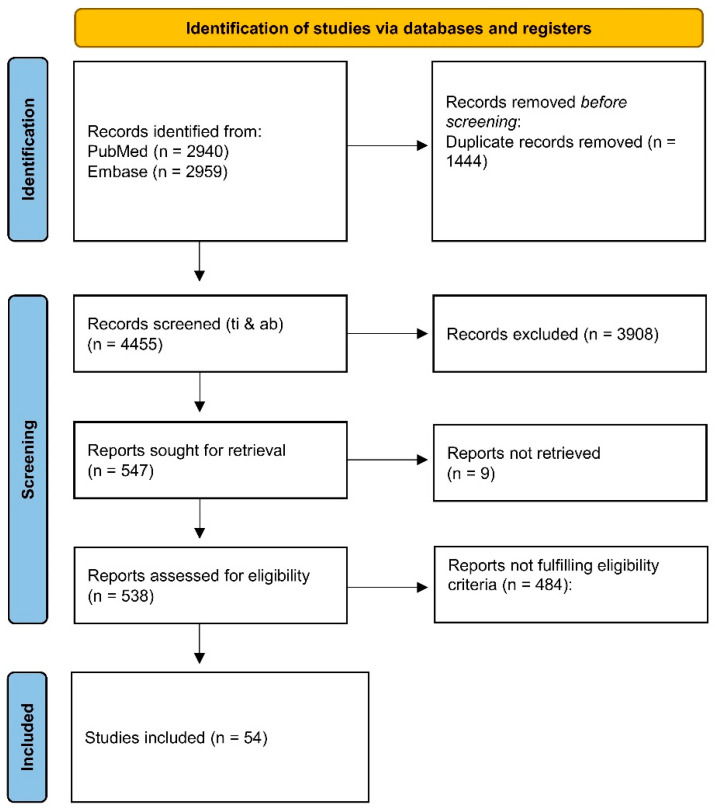
Preferred Reporting Items for Systematic Reviews and Meta-Analyses (PRISMA) flow chart. The diagram outlines the databases queried, the number of titles/abstracts screened, the full-text documents retrieved, and the number of included and excluded studies. Ti & ab, title and abstract [20].

We performed a systematic search of the literature available on Medline/PubMed and Embase in accordance with the Preferred Reporting Items for Systematic Reviews and Meta-Analyses (PRISMA) criteria [20] by combining the following keywords in a search string: antipsychotic, chlorpromazine, haloperidol, paliperidone, risperidone, asenapine, olanzapine, clozapine, quetiapine, amisulpride, aripiprazole, brexpiprazole, cariprazine, lurasidone, lumateperone, xanomeline, *Arc/Arg, BDNF, c-fos, fos, c-Jun, Jun, Egr1, Delta, Narp1, NPAS-4, Homer, Nor1, Nurr1, NGFI-B/Nur77, Nerve Growth Factor Inducible-B,* ISH, fMRI, EEG, functional connectivity, connectome, network. We registered the review protocol on the International Platform of Registered Systematic Review and Meta-Analysis Protocols (INPLASY2022110107). The search was conducted on 10 September 2022, and updates were checked until 14 October 2022. Hand-searching and checking the reference lists of the included articles turned up further papers. The search strings are available in the Supplementary Material. The search returned 5899 articles, which resulted in 4455 after removing duplicates. We deemed eligible English-written preclinical studies published in peer-reviewed journals that were pertinent to the topic, i.e., investigating the antipsychotic effects on synaptic architecture, IEGs expression, and, on a larger scale, anatomical and functional brain connectivity, without time or design methodology constraints. Although the names of the most widely tested antipsychotics in preclinical studies were used as keywords, others, selected from the generic term “antipsychotic”, also fit the inclusion criteria. EndNote X9 was used to manage all records. Two independent reviewers independently carried out screening and full-text assessment procedures (AB and GDS). Overall, 54 studies were included in the qualitative synthesis. The PRISMA flow diagram in Figure 1 reports the specifics of the search and selection procedures.

## 3. The Close Relationship between Brain Functional Connectivity and Postsynaptic Density

**Figure 2:**
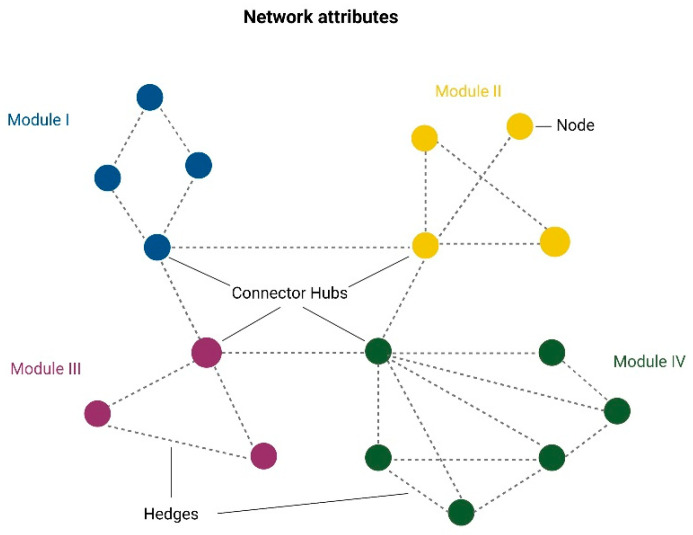
Graphical representation of network attributes. The circles indicate nodes; the nodes of the same color are grouped into modules; the dotted lines represent the hedges. Please, see the corresponding text for a full acknowledgment of network properties. Created with BioRender.com, accessed on 24 November 2022.

In recent years, the hypothesis that cognition and behavior rely on the neuronal capability to process and integrate information within and between specific brain network communities has attracted growing interest [21]. Network modules or communities are clusters of nodes that exhibit a highly correlated activity to attend to the same specialized tasks and are interlinked by hub regions [22]. Nodes represent the basic elements of networks, and their interconnections are depicted as weighted or unweighted, directed or undirected edges [22]. Individual nodes can show a low or high number of connections and, in the latter case, are called hubs [22]. A graphical representation of network attributes is shown in Figure 2. In brain networks, nodes are often but not exclusively used to provide topographical information on interregional patterns of activity and generally are representative of cortical and subcortical portions [23,24]. Given the relevance of brain networks in the expression of cognitive and behavioral phenotypes, it is not surprising that functional connectivity, also defined as functional networks, could have a major role in the understanding of the neurobiological correlates of schizophrenia and treatment-resistant schizophrenia [25,26,27]. 

While structural connectivity aims to identify white matter tracts that physically bridge brain regions, functional connectivity is involved in describing statistical co-activation patterns between different areas [28]. However, differences in mesoscale properties, such as nodes clustering, have been detected between functional and structural networks probably for the existence of polysynaptic connections not mediated by white matter fibers [28]. Specifically, while the analysis of correlations between the spontaneous blood oxygenation level-dependent (BOLD) signals in different brain regions allows for the identification of intrinsic connectivity in large-scale networks such as the default mode network (DMN), white matter tracts connecting parts of the parietal cortex and precuneus involved in the DMN have not been found [28]. Therefore, functional connectivity is not necessarily reflected by white fiber-mediated anatomical connections, emphasizing the importance of functional versus structural information in the study of psychotic disorders [28].

Accurate task-specific patterns of functional connectivity between large-scale brain circuits rest on well-organized processes of signal transmission and depend on synaptic integrity at the neuronal level [29]. In neurons, the information flow is conducted as an electrical signal along the axon, from dendrites to synapses, where a translation into a chemical message takes over [30]. An integral and functional synapsis is then required to spread the information flow over other neurons and allow efficient data processing in the whole central nervous system (CNS). In this framework, a balanced modulation of synaptic plasticity processes, through direct regulation of neuron–neuron interaction, is mandatory to orchestrate the correct topography of functional brain networks [18]. Specifically, neurotransmitter receptors, scaffold proteins, intracellular signaling molecules, and other elements comprised in the PSD, are essential to ensure proper communication between neurons [31]. Otherwise, altered synaptic plasticity may disrupt individual interactions among neurons, turning in functional dysconnectivity among entire brain regions [18]. 

Efforts to understand how synaptic changes can affect the overall brain activity as well as the connectivity of specific brain regions have pointed to the role of structural and functional rearrangement at the dendritic spine, where qualitative and quantitative alteration of PSD proteins (for instance, the NMDAR and the partner scaffold PSD-95) may modulate the synaptic strength, influence the reciprocal interaction between cluster of neurons (or of glia cells), and in turn impact the connectivity of functionally related brain regions [32]. 

Alterations in processes that regulate synaptic plasticity and PSD architecture may affect brain connectivity, disrupting the synchronized firing of those neurons organized in a high-frequency oscillation network (30–90 Hz/s), known as gamma waves, involved in cognition and memory functions [33,34,35]. Studies have associated altered gamma oscillations with neurological and psychiatric disorders, including Alzheimer’s disease, Parkinson’s disease, and schizophrenia [36,37]. Moreover, gamma oscillations were demonstrated to be modulated by antipsychotic treatment in preclinical studies [38,39,40]. In particular, risperidone and clozapine but not haloperidol were able to decrease phencyclidine (PCP)-induced prefrontal and cortical-hippocampal hypersynchrony without restoring hippocampal and circuit desynchronization probably responsible for their antipsychotic effect [40].

With regard to the pathophysiology of psychotic disorders, postmortem studies have unveiled significant differences in PSD proteins and presynaptic markers in schizophrenia patients compared to normal controls [41,42,43,44]. A computational model network was adopted to simulate how variability in excitatory synaptic strength between parvalbumin interneurons could regulate gamma power [44]. The variability in excitatory synaptic strength was computed by quantifying the vesicular glutamate transporter 1 (VGlut1) and PSD-95 protein levels in the postmortem brain of 20 matched pairs of controls and schizophrenia subjects [44]. 

Martin et al. reported the effects of olanzapine treatment on dysregulated gene expression in a non-human primate model of schizophrenia [45]. PCP-exposed primates showed abnormalities in 146 transcripts compared to controls, and olanzapine administration was able to reverse about the 34% of PCP-induced changes in gene expression [45]. In particular, several genes coding for synaptic and PSD proteins were found down-regulated in the pharmacological model of psychosis and enriched following antipsychotic treatment [45].

In this framework, schizophrenia has been conceptualized both as a synaptopathy and a dysconnectivity syndrome, in which improper processing of synaptic inputs and loss of signal integration between brain areas may account for psychopathological manifestations [17]. Moreover, a stable and robust re-organization of neuronal and regional interconnections in a pathological manner may support the persistence of psychotic symptoms [18]. The study of the effect of antipsychotics on functional brain connectivity and synaptic plasticity is, therefore, necessary to understand their real impact on the neurobiological core of clinical manifestations. By directly controlling confounding factors, preclinical models may provide further information on the molecular modifications induced by antipsychotics and their modulation of functional connectivity. Lastly, animal studies can help in developing translational models of prediction based on molecular parameters of postsynaptic density and interregional connections to foresee the efficacy and side effects of novel compounds. 

## 4. The Postsynaptic Density: A Molecular Hub Involved in Neuronal Communication and IEGs Expression

The PSD is an electron-dense thickening underneath the glutamatergic synapse [15] consisting of packed proteins forming a disc-shaped molecular platform whose width is a few hundred nm and whose thickness is ∼30–50 nm [46,47]. It consists of approximately 1500 molecules including neurotransmitter receptors (e.g., α-amino-3-hydroxy-5-methyl-4-isoxazolepropionic acid receptor—AMPAR-, N-methyl-D-aspartate receptor—NMDAR-, metabotropic glutamate receptors—mGluR-), adaptors and adhesion molecules (N-cadherin, neuroligins), and scaffolding proteins (e.g., Shank, Homer, PSD-95, GKAP) that have been proven to form condensates at physiological concentrations [16,48]. 

The PSD has been conceived as a structural and functional hub that is responsible for modeling the architecture of the dendritic spine and regulating the propagation of intracellular signals in response to various neuronal or environmental stimuli, resulting in modifications of synaptic plasticity and metaplasticity [49], and therefore relevant to the pathophysiology of several neuropsychiatric disorders [50]. Specifically, the role of PSD is to cluster postsynaptic receptors in proximity to released neurotransmitters to ensure a proper translation of the information flow through the synaptic cleft [51]. At the postsynaptic site, the activation of the glutamatergic receptors enhances multiple downstream signaling cascades, such as cAMP-dependent protein kinase (PKA), mitogen-activated protein kinase (MAPK), phospholipase C (PLC), calmodulin-dependent protein kinase II and IV (CaMKII/IV), phosphoinositide 3-kinase (PI3K), and Protein kinase B (PKB/Akt), that converge on cAMP-response element binding protein (CREB) phosphorylation [52,53]. The phosphorylation of CREB at Ser133 is mandatory to induce IEGs transcription, upon the binding to the cAMP response element (CRE) sequence of the promoters of its target genes and the recruitment of CREB-binding protein (CBP) [54].

IEGs comprise a large category of genes, whose expression is induced rapidly and transiently after postsynaptic activation and include, among others, *c-fos*, *zif268*, *brain-derived neurotrophic factor* (BDNF), *Narp*, *Homer1a*, and *Arc* [19]. Beyond their capability to fine-tune plastic and metaplastic processes involved in the regulation of long-term potentiation (LTP) and depression (LTD), IEGs represent a noteworthy molecular marker of synaptic integrity and postsynaptic activation due to their fleeting pattern of expression [19]. For the purposes of this paper, we dwelled on IEGs regional expression, with special regard to topographic changes induced by typical and atypical antipsychotics.

**Figure 3:**
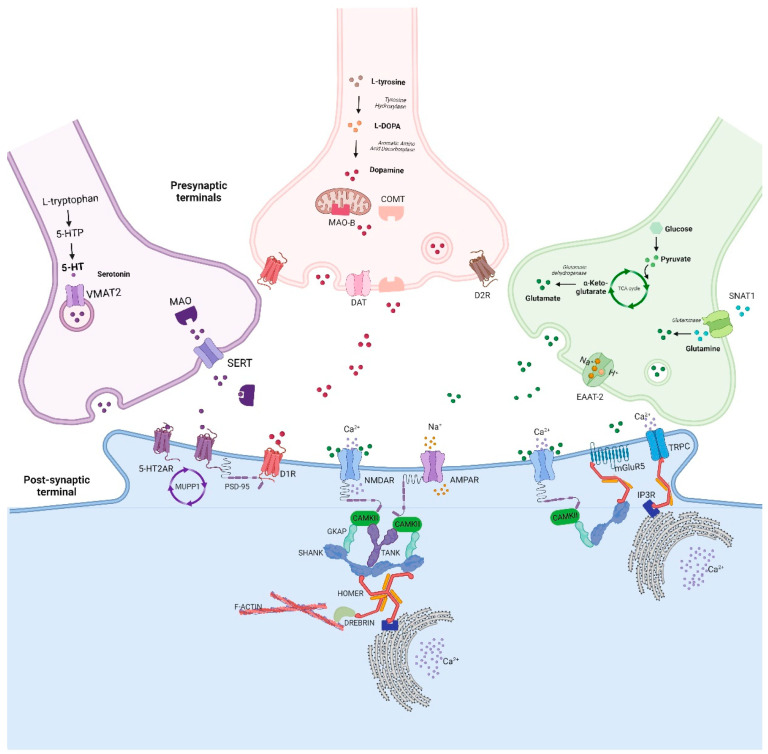
Interaction of presynaptic serotonergic, dopaminergic, and glutamatergic terminals with postsynaptic density proteins. 5-HTP, 5-Hydroxytryptophan; 5-HT, 5-hydroxytryptamine; VMAT2, Vesicular monoamine transporter 2; MAO, Monoamine oxidase; SERT, Serotonin transporter; L-DOPA, Levodopa; COMT, Catechol-O-methyltransferase; MAO-B, Monoamine oxidase B; DAT, Dopamine transporter; D2R, Dopamine receptor D2; TCA cycle, Tricarboxylic acid cycle; SNAT1, Sodium-coupled neutral amino acid transporters; EAAT-2, Excitatory amino acid transporter 2; 5-HT2AR, 5-hydroxytryptamine 2 A receptor; MUPP1, multiple PDZ protein 1; PSD-95, Postsynaptic density protein 95; D1R, Dopamine receptor D1; NMDAR, N-methyl-D-aspartate receptor; AMPAR, α-amino-3-hydroxy-5-methyl-4-isoxazolepropionic acid receptor; CAMKII, Ca^2+^/calmodulin-dependent protein kinase II; GKAP, Guanylate kinase-associated protein; TANK, TRAF Family Member Associated NFKB Activator; SHANK, SH3 and multiple ankyrin repeat domains protein; F-ACTIN, filamentous-actin; mGluR5, Metabotropic glutamate receptor 5; TRPC, Transient receptor potential channels; IP3R, Inositol trisphosphate receptor. Created with BioRender.com, accessed on 26 October 2022.

Over the well-known effect of glutamatergic neurotransmission on synaptic plasticity, other pathways have been variously related to PSD (Figure 3). In particular, the dopamine D1 receptor (D1R) was seen to interact directly with the NMDA receptor subunits 1 (NR1) and indirectly with the NMDAR by the interposition of PSD-95 (Figure 3) [55]. Moreover, the serotonergic 2A receptor (5-HT_2A_R) was found to physically engage with multiple PDZ protein-1 (MUPP1) and PSD-95 at the dendritic spines of cortical pyramidal neurons (Figure 3) [56]. Therefore, not only glutamatergic but also serotonergic and dopaminergic pathways, as well as adrenergic and muscarinic receptors, can interact and modulate the composition of the PSD, with consequent changes in processes that regulate synaptic plasticity and interneural communicability [57,58]. Thus, typical and atypical antipsychotics through individual pharmacodynamic profiles could diversely impact PSD changes and exhibit specific spatial effects on brain regions. This issue appears to be particularly relevant for defining antipsychotics-based differences in the modulation of interregional functional connectivity.

Aberrant processes of assembling and disassembling PSD structures have been conceptualized as the possible neurobiological basis for psychosis as well as for the delivery of antipsychotic effects. In fact, multiple lines of evidence have suggested that psychiatric disorders, including schizophrenia and affective disorders, may be related to the disruption of synaptic structure, function, and capability to make connections between different brain regions [59,60,61]. In this regard, a recent meta-analysis examined postsynaptic elements in the postmortem brain tissue of patients affected by schizophrenia compared to healthy controls and found an overall decrease in dendritic spine density (DSD) in cortical but not subcortical regions [13]. A significant reduction in the PSD-95 and mGluR1protein levels as well as a decrease in the ratio between Homer1b/c and Homer1a isoforms were found in the hippocampus of schizophrenia patients compared to controls [62]. Other studies proved an alteration of PSD-95 protein expression also in the dorsolateral prefrontal cortex (DLPFC) of patients with schizophrenia [63,64]. Through the application of a machine learning-based algorithm, a recent postmortem study identified multiple clusters of molecular markers of schizophrenia, including several proteins involved in the regulation of synaptic plasticity processes [41]. The above-mentioned defects detected in the PSD of psychotic patients and even others, such as the loss of neuropil, reduced cell size and synaptic counts, and a decrease in the protein expression of synaptophysin, strongly support the neurodevelopmental hypothesis for schizophrenia [14]. Overall, alterations in molecular components of the PSD in schizophrenia patients could result in an improper clustering of postsynaptic receptors, with a following impairment in the neuron–neuron interaction and whole-brain functional connectivity.

The extent to which antipsychotics modulate receptor activity is essential to ensure proper processes of neurite outgrowth, synaptogenesis, and spine organization [65]. In this framework, antipsychotics have shown a different capability to regulate PSD composition and spine architecture by exhibiting different pharmacodynamic profiles and acting through epigenetic mechanisms [65,66,67,68,69]. Specifically, aripiprazole and clozapine were found to increase PSD-95 protein levels, the number of dendritic spines, and the number of PSD puncta in cortical neuron cultures [70]. Otherwise, haloperidol had opposite effects on synaptic architecture by reducing PSD-95 levels, the number of spines, and the number of PSD puncta [70]. In another study, the administration of haloperidol, but not clozapine, was associated with an increase in the number of perforated synapses, which are characterized by a discontinuous density when observed in transversal sections, within the caudate nucleus [71]. On the other hand, haloperidol showed to significantly reduce the average volume of dendritic spines in layer VI neurons of the PFC, whereas clozapine increased the number of symmetric axospinous synapses [72]. Furthermore, olanzapine and aripiprazole, but not haloperidol, increased PSD-95 protein levels in rat hippocampal neuronal cultures under both normal and toxic conditions [73]. In prefrontal cortical neurons, olanzapine was shown to prevent PCP-induced reduction in neurite length and branches as well as the decrease in both synaptophysin and PSD-95 mRNA and protein levels through a neuregulin-mediated mechanism [74]. The application of the D2R-specific agonist quinpirole impaired the synaptic architecture of cortical neuron cultures by reducing spine branches and length, along with a decrease in PSD-95 and synaptophysin levels, especially in animals showing a dysfunctional copy of the disrupted schizophrenia 1 (DISC1) gene [75]. In the same study, the pre-treatment with aripiprazole and haloperidol significantly prevented the quinpirole-induced changes in spine morphology, with a more effective impact exerted by aripiprazole [75]. Interestingly, the two antipsychotics could act through different intracellular signaling, with aripiprazole, but not haloperidol, able to increase pAkt-Thr308 levels in cortical neurons [75]. In monocyte-derived-neuronal-like cells (MDNCs) isolated from schizophrenia patients and healthy controls, incubation with haloperidol showed no capability to modulate the structure and the pruning of primary and secondary neurites [76]. Therefore, the effects of antipsychotics on synaptic architecture could depend on the specific brain region examined, with relative differences between cortical and subcortical areas [71,72,73]. In addition, antipsychotics could restore, at least in part, a working pattern of brain functional connectivity through the regulation of the synaptic architecture and spine morphology as well as PSD composition and IEGs expression, with differences between typical and atypical compounds [65,70,71,72,73,75].

**Figure 4:**
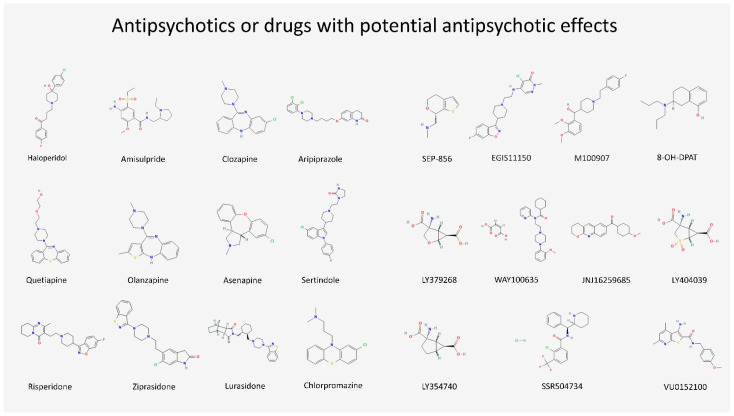
Graphical representation of the chemical structure of all antipsychotics and drugs with potential antipsychotic effects discussed in the present review.

Given their role as markers of postsynaptic activity, IEGs may represent a remarkable candidate to investigate how acute or chronic antipsychotics administration could be responsible for setting the molecular framework for long-term changes at the PSD level, resulting in clinical phenotypes, brain morphological variations, and treatment responses [77,78,79]. In addition, we represented, in Figure 4, the chemical structure of all antipsychotics and drugs with potential antipsychotic effects discussed in the present review by consulting the PubChem database [80].

## 5. Antipsychotics-Mediated IEGs Regulation: Toward a Topographical Analysis of Genes Expression Patterns

Several studies attempted to explore the effect of individual antipsychotics on the IEGs spatial pattern of expression in a preclinical setting. Behind their molecular tasks and their capability to influence synaptic plasticity and metaplasticity, IEGs expression was used specifically to obtain topographical information on regional brain activation. We gained evidence on antipsychotics-induced increase or decrease in IEGs expression, with regard to time of exposure, region of interest, and type of IEG involved, as shown in Table 1. Antipsychotic action on PSD proteins’ assembly and interaction may trigger changes in dendritic spines and therefore influence trans-synaptic communication between neurons [70,81].

Multiple differences were found in the capability of individual molecules to modulate neuronal activity in the animal brain, especially between typical and atypical antipsychotics.

Of interest, haloperidol, a first-generation antipsychotic with a strong affinity for the D2R [82], was found to regulate striatal activity by increasing the expression of IEGs, such as *Homer1a*, *Arc*, *c-fos, zif-268*, *Ania-3*, and *Nurr77* in both an acute and chronic paradigm [79,83,84,85,86,87,88,89,90,91,92,93,94,95]. Inconsistent evidence linked haloperidol activity with the modulation of IEGs in cortical regions [96], showing a decrease in *Homer1a*, *Arc*, and *Nurr77* expression in the somatosensory, cingulate, insular, motor, and medial agranular cortices [79,92,93,97] and an increase of *Nurr77* and *Krox-20* in the PFC [83,88] and of *zif-268* and *Homer1a* in the somatosensory, cingulate, insular, motor, and medial agranular cortices [85,86,94].

Amisulpride, an “atypical” second-generation antipsychotic, that is almost a pure D2/D3R antagonist with relevant presynaptic action, was found to effectively treat both positive and, even if with less degree, negative symptoms in psychosis, inducing only a few extrapyramidal side effects through the capability to enhance dopaminergic neurotransmission [98]. In preclinical studies, the acute administration of amisulpride was shown to increase IEGs expression in both cortical and subcortical regions [85,94].

Three atypical antipsychotics, risperidone, lurasidone, and ziprasidone, were found to increase the expression of *arc*, *zif-268*, *Homer1a*, and *c-fos* in the striatum, hippocampus, PFC, cingulate cortex, and other cortical regions, with specific patterns of expression depending on the delivered dose [86,99,100,101,102].

Repetitive administrations of quetiapine, olanzapine, and clozapine were associated with an overall increase in *BDNF* expression, especially in the hippocampus and PFC [103,104,105,106,107,108]. Otherwise, acute administrations of clozapine and olanzapine demonstrated the capability to modulate both cortical and striatal regions and induced a heterogenous pattern of IEGs modifications, based on the specific gene regulated, the dose, and the brain area involved [79,83,86,87,88,89,90,91,93,97,99,109].

In addition, asenapine also was found to differently modulate IEGs expression according to the time of exposure. Specifically, an elevation in *c-fos*, *zif-268*, and *Homer1a* was detected in the striatum and motor, insular, somatosensory, medial agranular, and cingulate cortices after acute injection, whereas chronic administration was associated with a decrease in *Homer1a* and *arc* expression in cortical regions and an increase in the striatum [79,93].

The chronic administration of aripiprazole, a third-generation antipsychotic with a D2R partial agonism, showed to reduce *BDNF* expression in the hippocampus and, simultaneously, raise *arc* mRNA levels in the striatum and PFC [110].

A new compound with antipsychotic effects, SEP-363856 (SEP-856), was tested in a preclinical setting to define the spatial profile of IEGs induction [111]. SEP-856 was found to increase the expression of *Arc*, *c-Fos*, *Zif-268*, and *Npas4* in the hippocampus and PFC through a possible agonism at 5-HT_1A_R and trace amine-associated (TAAR1) receptors [111].

A comprehensive summary of the specific IEGs expression pattern induced by single antipsychotics is provided in Table 1, with consideration of the brain region affected, the administration paradigm, and the type of IEG included.

**Table 1:** The topographical pattern of IEGs expression induced by antipsychotics.

Antipsychotic Drug	Administration	Authors	Brain Region	IEGs Expression
Amisulpride	Acute	de Bartolomeis et al., 2013 [85]	Caudate-Putamen	↑ *c-fos,* ↑ *Arc,* ↑ *Homer1a,* ↑ *Zif-268*
			Cingulate Cortex	↑ *Zif-268*
			MC	↑ *Zif-268,* ↓ *Ania-3*
		de Bartolomeis et al., 2016 [94]	SS, Cingulate Cortex, M2, MC, NAc, Caudate-Putamen	↑ *Homer1a*
Aripiprazole	Chronic	Luoni et al., 2014 [110]	Hippocampus	↓ *BDNF*
			PFC, Striatum	↑ *Arc*
Asenapine	Acute	de Bartolomeis et al., 2015 [79]	Caudate-Putamen, NAc Shell and Core	↑ *Arc,* ↑ *c-fos,* ↑ *zif-268,* ↑ *Homer1a*
			SS, IC	↑ *Homer1a*
			M2, MC, Cingulate cortex	↑ *Homer1a,* ↓ *Arc*
	Chronic	Buonaguro et al., 2017 [93]	Striatum	↑ *Homer1a,* ↑ *Arc*
			IC, M2, MC, SS	↓ *Homer1a,* ↓ *Arc*
Clozapine	Acute	Beaudry et al., 2000 [83]	PFC, Cingulate Cortex, NAc Shell, Dorsolateral Striatum	↑ *Nurr77*
		Nguyen et al., 1992 [90]	Striatum	↑ *Zif-268*
		MacGibbon et al., 1994 [89]	Striatum, NAc, Ventral Septum	↑ *c-fos,* ↑ *Jun B,* ↑ *Krox24,* ↑ *FRA*
		Langlois et al., 2001 [97]	SS	↑ *Nurr77*
		Cochran et al., 2002 [84]	PFC	↑ *c-fos,* ↑ *fos B,* ↑ *fra2*
			Mediodorsal thalamus	↑ *c-fos*
		Polese et al., 2002 [87]	NAc	↑ *c-fos,* ↑ *Homer1a*
			Caudate-Putamen	↑ *Homer1a*
		Werme et al., 2000 [91]	NAc Shell	↑ *c-fos,* ↑ *Nor1,* ↑ *Nurr77*
		Robbins et al., 2008 [88]	NAc	↑ *c-fos*
			Hippocampus	↓ *c-jun*
			Striatum	↑ *c-fos,* ↑ *sgk-1,* ↓ *Narp*
			Thalamus	↑ *c-fos,* ↓ *arc*
		Pinna et al., 2019 [99]	PL, IL, PFC	↑ *c-fos*
	Chronic	Kontkanen et al., 2002 [105]	PFC, FC, Cingulate Cortex	↑ *c-fos,* ↑ *c-jun,* ↑ *junB*
		Kim et al., 2012 [108]	Whole brain	↑ *BDNF*
		Verma et al., 2007 [106]	PFC	↑ *c-fos*
		Rizig et al., 2012 [103]	Whole brain	↑ *BDNF*
		Bai et al., 2003 [104]	Hippocampus	↑ *BDNF*
Haloperidol	Acute	Beaudry et al., 2000 [83]	PFC, NAc Shell, Dorsolateral Striatum	↑ *Nurr77*
		Cochran et al., 2002 [84]	Mediodorsal thalamus	↑ *c-fos*
			NAc Shell	↑ *fra2*
		de Bartolomeis et al., 2015 [79]	NAc Shell and Core, Caudate-Putamen, Lateral septum	↑ *Homer1a,* ↑ *Arc,* ↑ *c-fos,* ↑ *zif-268*
			SS, M2, MC, Cingulate cortex, IC	↓ *Arc*
		Robbins et al., 2008 [88]	Thalamus	*↑ fra-1, ↓ BDNF*
			Striatum	↑ *c-fos*, ↑ *Krox-20,* ↑ *arc*
			NAc Shell and Core	↑ *c-fos*, ↑ *Krox-20,* ↑ *arc,* ↓ *c-jun*
			PFC	↑ *Krox-20*
		De Bartolomeis et al., 2013 [85]	Cingulate Cortex	↑ *Homer1a*
			Caudate-Putamen, NAc Shell and Core	↑ *Ania-3,* ↑ *c-fos,* ↑ *Homer1a,* ↑ *Zif-268,* ↑ *Arc,* ↑ *Norbin*
			IC, MC, SS	↑ *Zif-268*
		Iasevoli et al., 2010 [95]	Caudate-Putamen, NAc Shell and Core	↑ *Ania-3,* ↑ *arc,* ↑ *Homer1a*
			Cingulate Cortex, M2	↑ *Ania-3,* ↑ *Homer1a*
			MC, SS, IC	↑ *Homer1a*
		De Bartolomeis et al., 2016 [94]	Caudate-Putamen, MC	↑ *Homer1a*
		MacGibbon et al., 1994 [89]	Striatum, NAc, Ventral Septum	↑ *c-fos,* ↑ *Jun B,* ↑ *Krox24,* ↑ *FRA*
		Nguyen et al., 1992 [90]	Striatum	↑ *Zif-268,* ↑ *c-fos*
		Polese et al., 2002 [87]	NAc, Caudate-Putamen	↑ *c-fos,* ↑ *Homer1a*
		Langlois et al., 2001 [97]	SS	↓ *Nurr77*
		Werme et al., 2000 [91]	NAc Shell and Core, Caudate-Putamen	↑ *c-fos,* ↑ *Nor1,* ↑ *Nurr77*
		Dell’Aversano et al., 2009 [92]	Caudate-Putamen, NAc Core	↑ *Ania-3,* ↑ *Homer1a*
			Parietal Cortex	↓ *Homer1a*
		Iasevoli et al., 2010 [86]	Striatum	↑ *Homer1a*
		Iasevoli et al., 2010 [86]	Striatum	↑ *Homer1a*
	Transient treatment	Samaha et al., 2008 [112]	Striatum	↑ *c-fos*
	Chronic	Buonaguro et al., 2017 [93]	Caudate-Putamen, NAc	↑ *Homer1a*
			IC, MC, M2, SS	↓ *Homer1a,* ↓ *Arc*
		Fumagalli et al., 2009 [109]	Striatum	↓ *Arc*
		Verma et al., 2007 [106]	PFC	↓ *c-fos,* ↑ *Zif-268*
		Iasevoli et al., 2010 [86]	Striatum, Cingulate Cortex, MC, M2, SS, IC	↑ *Homer1a*
		Iasevoli et al., 2010 [86]	Caudate-Putamen, NAc	↑ *Homer1a,* ↑ *Ania-3*
			SS, MC, M2, Cingulate Cortex	↑ *Homer1a*
		Parikh et al., 2004 [113]	Hippocampus	↓ *BDNF*
Lurasidone	Acute	Luoni et al., 2014 [101]	Striatum	↑ *Arc,* ↑ *zif-268*
			Hippocampus	↑ *Arc,* ↓ *Npas4*
	Chronic	Luoni et al., 2014 [101]	Hippocampus, PFC, Striatum	↑ *Arc,* ↑ *zif-268*
		Fumagalli et al., 2012 [102]	PFC, Hippocampus	↑ *BDNF*
Olanzapine	Acute	De Bartolomeis et al., 2015 [79]	M2, MC, Cingulate cortex, IC	↑ *Homer1a*
			Caudate-Putamen, NAc Shell	↑ *c-fos,* ↑ *zif-268,* ↑ *Homer1a*
		Iasevoli et al., 2010 [86]	Striatum	↑ *Homer1a*
		Pinna et al., 2019 [99]	BSTL, IPAC, SLEA, CeA	↑ *c-fos*
		Buonauguro et al., 2017 [93]	Striatum, NAc Shell	↑ *Homer1a,* ↓ *Arc*
			SS, IC, MC, M2, Cingulate Cortex	↓ *Arc*
		Fumagalli et al., 2009 [109]	Striatum, FC	↓ *Arc*
	Chronic	Bai et al., 2003 [104]	Hippocampus	↑ *BDNF*
Quetiapine	Chronic	Park et al., 2006 [107]	Hippocampus	↑ *BDNF*
Risperidone	Acute	Robinet et al., 2001 [100]	Striatum	↑ *c-fos*
		Iasevoli et al., 2010 [86]	Striatum	↑ *Homer1a*
		Pinna et al., 2019 [99]	PL, IL, PFC	↑ *c-fos*
	Chronic	Robinet et al., 2001 [100]	Striatum	↑ *c-fos*
Sertindole	Acute	Iasevoli et al., 2010 [95]	SS, IC	↑ *Homer1a*
Ziprasidone	Acute	Iasevoli et al., 2010 [86]	Striatum, SS, MC, Cingulate cortex, IC	↑ *Homer1a*
	Chronic	Iasevoli et al., 2010 [86]	Striatum, MC, IC	↑ *Homer1a*
SEP-856	Acute	Begni et al., 2021 [111]	PFC	↑ *Arc*, ↑ *c-Fos*, ↑ *Egr1*, ↑ *Npas4*
			Hippocampus	↑ *Arc*, ↑ *c-Fos*

↑ = Gene expression is up-regulated; ↓ = gene expression is down-regulated; BSTL = bed nucleus of stria terminalis lateral division; CeA = central amygdaloid; FC = frontal cortex; IC = insular cortex; IL = infralimbic; IPAC = interstitial nucleus of the posterior limb of the anterior commissure; M2= medial agranular cortex; MC = motor cortex; NAc = nucleus accumbens; PFC = prefrontal cortex; PL= prelimbic; SLEA = sublenticular extended amygdale; nucleus; SS = somatosensory cortex.

## 6. Methods to Study Brain Functional Connectivity in Translational Models: A Brief Overview of Pros and Cons

Even though there are several methods to explore the impact of antipsychotic drugs on brain functional connectivity in preclinical models, two are the most widely applied: functional magnetic resonance imaging (fMRI) and, on the other hand, electrophysiology/electroencephalogram (EEG), which assess interregional patterns of co-activation and neuronal synchrony by measuring brain blood flow and electrical activity, respectively [114,115,116].

However, these methods show several limitations. First, fMRI measures regional differences in cerebral blood flow, indirectly reflecting presynaptic activity and not postsynaptic modifications, which are more representative of those neuroplastic processes altered in schizophrenia [117]. In addition, fMRI acquisitions can be affected by small head movements and often require sedation [118]. On the other hand, sedative drugs can significantly affect functional connectivity and provide different patterns of co-activation depending on the specific compounds adopted, thus compromising the consistency and reproducibility of individual studies [118]. Although feasible in multiple settings and characterized by elevated temporal resolution, EEG applications may suffer from reduced topographical resolution with difficulty in detecting the exact location where the activity is generated and are characterized by a poor signal-to-noise ratio, which severely impairs the sensitivity and specificity of the data [119].

Moreover, human studies have difficulties in exploring the effect of different antipsychotics on network connectivity, and antipsychotic treatment is rather considered a confounding variable. Except for research conducted on drug-naive participants, it may be challenging to distinguish between the structural and functional changes induced by antipsychotic agents and those attributable to the disease itself. In this context, preclinical research may enable us to address these shortcomings, by analyzing changes in functional connectivity after pharmacological challenges in animals.

To overcome these limitations, two studies explored changes in functional connectivity after antipsychotic administration by directly assessing IEGs expression through an in situ hybridization (ISH) protocol and generating group-specific correlation matrices to obtain details on differential patterns of co-activation between brain areas [8,9]. The simultaneous detection of molecular markers of postsynaptic activity and spatial information offers a unique opportunity to relate alterations in neuronal plasticity to connectivity dysregulation and represents a novel method for testing the effectiveness of antipsychotics in preclinical models.

Furthermore, novel molecular techniques, such as ISH chain reaction (HCR), will probably enable a step forward in the research of the neurobiology underlying psychiatric disorders and help shed light on antipsychotics’ mechanism of action due to the possibility of matching topographical information with transcriptomic or proteomic data [120].

## 7. Antipsychotics-Mediated Modulation of Functional Connectivity: Preliminary Evidence from Preclinical Studies

Several antipsychotics and even new molecules in experimentation were tested in preclinical models to assess their putative impact on functional connectivity, especially but not exclusively in brain networks disrupted by the injection of drugs that induce a psychotic-like phenotype, as outlined in Table 2.

Of interest, the administration of N-methyl-D-aspartate receptor (NMDAR) antagonists such as PCP, MK-801, and ketamine [121,122] to properly model manifestations resembling schizophrenia in animals determined a disconnection between and within the neuronal activity of the hippocampus and prefrontal cortex (PFC). PCP was found to induce different patterns of brain activity depending on its levels, with high doses globally impairing functional connectivity and low doses affecting mainly the hippocampal and frontal regions [123]. The alteration in the hippocampal-PFC pathway is particularly relevant to the physiopathology of schizophrenia, as this pathway is involved in the regulation of learning, behavioral flexibility, novelty processing, and memory and has been found to be impaired in patients with schizophrenia [124,125,126,127].

Two electrophysiological studies found an overall dysregulation in the activity of pyramidal cells of the PFC in rats and mice treated with PCP, including alterations in the neuronal firing rate, reduced cortical synchrony, and a decrease in slow cortical oscillations [128,129]. In both studies, the co-administration of antipsychotics was effective in reversing PCP-induced modifications. Precisely, haloperidol and clozapine were both able to counteract the alterations in pyramidal cell firing and the loss in cortical synchrony [128]. By the complementary application of histochemistry techniques, clozapine was also seen to prevent the PCP-mediated induction of c-fos levels in pyramidal neurons of the PFC, showing the capability to modulate the glutamatergic output and IEG expression [128]. Clozapine ability to regulate the neuronal activity in the PFC has been ascribed to its partial agonism at the serotonin 1A receptor (5-HT_1A_R), as highlighted by the use of mice knockout for the serotonin receptor [129]. Similarly, chlorpromazine, a first-generation antipsychotic, was effective in preventing the excitatory action of ketamine on the EEG bands of the hippocampus, with an even more pronounced effect after the addition of alpha-lipoic acid [130]. These findings were supported by a recent study, in which haloperidol, clozapine, and risperidone effectively prevented the PCP-induced desynchronization of the PFC in rats [127]. In the same study, the effect of a novel compound EGIS-11150, an inhibitor of adrenergic alpha1 and alpha2c, and serotonergic 5-HT_2A_R and 5-HT_7_R receptors, were evaluated after both PCP and ketamine administrations [127]. EGIS-11150 not only showed to fully tackle the EEG modifications induced by ketamine and PCP but also to increase the hippocampus-PFC coherence and reverse the inhibition of long-term potentiation (LTP) induced by stressors more than clozapine and haloperidol [127].

Moreover, the effects on brain network organization may be quite different between typical and atypical antipsychotics, with selective neural mechanisms underlying drugs’ therapeutic action [40,131]. In a murine model of psychosis, the PCP injection was responsible for an increase and decrease in the neuronal synchronization of the PFC and hippocampus, respectively, with an impairment in the overall circuit connectivity [40]. These alterations along with pathological behavioral phenotypes were partially reversed by atypical but not typical antipsychotics through a presumptive major activity on cortical serotonergic receptors [40,131,132,133]. These results were replicated by the application of an in vivo amperometry technique in a ketamine-induced psychotic-like model, showing the ability of clozapine but not haloperidol to restore a physiological pattern of the O_2_ signal in the PFC and medial ventral striatum [134]. In another study, the mid-dose of atypical antipsychotics with stronger affinity to 5-HT_2A_, 5-HT_2C_, and 5-HT_1A_ receptors showed a positive association with increased striatal–PFC functional connectivity, whereas the affinity for the D2R was not related to any connectivity measure, as detected by fMRI application [135]. However, haloperidol, a strong D2R blocker, was effective in modulating functional connectivity between the substantia nigra and several brain regions, including the cingulate and prefrontal cortices, hippocampus, ventral pallidum, and motor cortex, as detected by fMRI [136]. These results were partially confirmed by an ISH study assessing *Homer1a* gene expression in 33 regions of interest (ROIs) and performing a network analysis, which showed reduced intercorrelations between brain areas, such as cingulate cortex, anterior insula, caudate-putamen, and nucleus accumbens, as well as enhanced interactivity between cortical and striatal regions, and within caudate putamen subdivisions [8]. A previous study assessed the expression of *zif268*, an IEG, in 83 ROIs after haloperidol administration via an ISH protocol and found increased intercorrelations between the dorsolateral striatum and thalamus, and between different subdivisions of the thalamus [9]. Taken together, these results highlight the ability of compounds with different pharmacodynamic profiles to induce specific patterns of brain activation, with a stronger effect of atypical antipsychotics compared to typical ones on PFC connectivity via the modulation of serotonergic receptors in disrupted networks.

Beyond the serotonergic modulation of hippocampal and PFC activity, further evidence pointed to the glutamatergic pathway as a key regulator of brain functional connectivity. Specifically, the increase in cortical gamma band oscillations observed after the administration of MK-801, an NMDAR antagonist, was reversed not only by haloperidol and clozapine but also by riluzole, a glutamate release inhibitor, and LY379268, a mGlu2/3R agonist [137]. In another study, the detrimental effects on brain networks due to 5-methoxy-N,N-dimethyltryptamine (5-MeO-DMT), a natural hallucinogen contained in Ayahuasca, were evaluated through a combined electrophysiology and fMRI protocol and included dysregulation of pyramidal neuron activity and reduced power of low-frequency cortical oscillations in rodent PFC, which were partially reversed by haloperidol, clozapine, risperidone, and even LY379268, a mGlu2/3R agonist [138]. Additional evidence pointed to mGlu2/3R agonists as putative regulators of brain functional connectivity. In this regard, other studies highlighted the ability of mGlu2/3R agonists, such as TASP0443294, LY379268, and LY404039, to partially increase EEG coherence and counteract the basal gamma hyperactivity detected in the frontal regions of rats treated with NMDAR antagonists [139,140,141,142,143]. To differentiate the impact of mGlu2Rs and mGlu3Rs on brain functional networks, Wood and colleagues employed Han Wistar rats, which lack mGlu2R but not mGlu3R [144]. The mGlu2/3R agonist, LY354740, showed to reverse psychotic-like behaviors induced by amphetamine and PCP administration in control Wistar but not in Han Wistar rats. In the same experimental setting, the mGlu2/3R agonist LY379268 differentially modified the network oscillatory activity, reducing the power of high-frequency gamma bands in control Wistar but not in Han Wistar rats [144]. In addition, regulators of the NMDAR have been experimented in clinical trials and may account for beneficial antipsychotic effects also by modulating brain connectivity [6]. Specifically, SSR504734, an inhibitor of the glycine transporter-1 (GlyT1) could enhance the NMDAR-mediated glutamatergic neurotransmission by increasing extracellular glycine levels and was shown to significantly reduce the ketamine-induced increase of brain metabolic activity in the PFC, cingulate cortex, hippocampus, nucleus accumbens, and anteroventral thalamic nucleus [145]. Moreover, SSR504734 reversed the increase of the absolute power of the alpha1 band in rats treated with MK-801, together with a reduction in psychotic-like behaviors [145]. Overall, these findings suggest a strong impact of mGlu2R and NMDAR modulators on brain functional connectivity with a presumptive antipsychotic effect in a translational and clinical context.

Further, antipsychotic effects were reported after the administration of VU0152100, a positive allosteric modulator of the M4 muscarinic receptor, which reverted amphetamine-mediated psychotic-like behaviors and, simultaneously, tuned regional patterns of functional connectivity in the nucleus accumbens, retrosplenial cortex, motor cortex, hippocampus, and medial thalamus [146].

For a comprehensive and systematized insight into the overall topographical modifications of brain activity induced by single antipsychotics, please see Table 2.

**Table 2:** Antipsychotics modulation of brain functional connectivity in a preclinical setting.

Author	Methods	Antipsychotics	Results
Kargieman et al., 2007 [128]	Electrophysiology, ISH (*c-fos*)	Haloperidol, Clozapine	Haloperidol and clozapine reversed PCP-induced alterations in pyramidal cell firing and the loss in cortical synchrony of limbic PFC. Clozapine prevented the PCP-mediated induction of *c-fos* levels in pyramidal neurons of the PFC.
Kargieman et al., 2012 [129]	Electrophysiology	Clozapine	Clozapine counteracted the PCP-mediated reduction in slow cortical oscillations in the PFC of both wild-type and 5-HT_2A_R knockout mice but not of 5HT_1A_R mice.
Sampaio et al., 2017 [130]	EEG	Chlorpromazine (+/− alpha-lipoic acid)	Chlorpromazine prevented the ketamine-induced increase in the average spectral power of gamma low-bands. The combination of chlorpromazine with alpha-lipoic acid potentiated the inhibitory effects on gamma low-band oscillations.
M. Spedding et al., 2022 [127]	EEG	Clozapine, Risperidone, Haloperidol, EGIS 11150	EGIS 11,150 increased Hippocampal-PFC coherence and the 8–9 Hz theta band of the EEG power spectrum. Clozapine, risperidone, haloperidol, and EGIS 11,150 prevented the desynchronization effect of PCP or ketamine on the power spectra of the PFC.
C. Delgado-Sallent et al., 2021 [40]	EEG	Risperidone, Clozapine, Haloperidol, M100907, 8-OH-DPAT	PCP increased PFC synchronization, impaired hippocampal synchronization, and disrupted circuit connectivity. Risperidone and clozapine, but not haloperidol, reduced PCP-induced prefrontal hypersynchrony whereas none of the substances restored hippocampal and circuit desynchronization. Also, 5-HT_2A_R antagonism by M100907 and 5-HT_1A_R agonism by 8-OH-DPAT were effective in reducing prefrontal hypersynchrony.
Gener et al., 2019 [131]	EEG	Risperidone, 8-OH-DPAT, M100907, Haloperidol	Risperidone, 8-OH-DPAT, M100907, and haloperidol reduced locomotor activity of mice along with suppression of neural spiking, power of theta and gamma oscillations in PFC and hippocampus, and reduction of PFC-Hippocampal theta phase synchronization.
Sebban et al., 2002 [132]	EEG	Clozapine, M100907	Clozapine and M100907 antagonized the decrease in power between 5 and 30 Hz but not the increase in power at 1–3 Hz in PFC caused by PCP.
Feinberg et al., 1998 [133]	EEG	Haloperidol	Haloperidol potentiates the EEG slowing of MK-801 but it blocks its motor effects and its neurotoxic vacuolization.
Jennifer Li et al., 2014 [134]	Electrophysiology	Clozapine, Haloperidol	Clozapine but not haloperidol attenuated the ketamine-induced increase in O_2_ signal and PFC-mVS coherence.
F. Tollens et al., 2018 [135]	fMRI	Amisulpride, Risperidone, Olanzapine	The mid-dose of antipsychotic compounds with stronger affinity to serotonin 5-HT_2A_R, 5-HT_2C_R, and 5-HT_1A_R was associated with increased PFC-striatal functional connectivity, whereas no correlation was observed for D2R.
N. Gass et al., 2013 [136]	fMRI	Haloperidol	Haloperidol reduced the functional connectivity between the substantia nigra and several brain regions, including the cingulate cortex, PFC, posterodorsal hippocampus, ventral pallidum, and motor cortex.
A. Barone et al., 2021 [8]	ISH (*Homer1a*)	Haloperidol	Haloperidol reduced intercorrelations between brain areas, such as the cingulate cortex, anterior insula, caudate-putamen, and nucleus accumbens, as well as enhanced interactivity between cortical and striatal regions, and within caudate putamen subdivisions.
A. L. Wheeler, 2014 [9]	ISH (*zif268*)	Haloperidol	Haloperidol increased intercorrelations between the dorsolateral striatum and thalamus, and between different subdivisions of the thalamus.
T. Hiyoshi et al., 2014 [137]	EEG	Haloperidol, Clozapine, Riluzole, LY379268	Haloperidol, Clozapine, Riluzole, and LY379268 reversed the increase in cortical gamma band oscillations observed after the administration of MK-801.
S. Riga et al., 2014 [138]	Electrophysiology/fMRI	Haloperidol, Clozapine, Risperidone, LY379268, M100907, WAY100635	The natural hallucinogen 5-MeO-DMT increased the firing rate of 51% of neurons and reduced that of 35% of pyramidal cells of the PFC as well as decreased the BOLD signal in the PFC and V1. Clozapine and haloperidol reversed the effects on the pyramidal discharge of 5-MeO-DMT. Haloperidol, Clozapine, Risperidone, LY379268, M100907, and WAY100635 antagonized the reduction in the power of low-frequency cortical oscillations induced by 5-MeO-DMT.
H. Hikichi et al., 2015 [139]	Electrophysiology	TASP0443294	TASP0443294 inhibited methamphetamine-induced hyperlocomotion in rats and reduced the basal gamma hyperactivity in the PFC exerted by ketamine.
T. Hiyoshi et al., 2014 [140]	Electrophysiology	LY379268, JNJ16259685	LY379268 and JNJ16259685 decreased the ketamine-induced basal gamma hyperactivity in the rat frontal cortex.
A. Ahnaou et al., 2016 [141]	Electrophysiology	Risperidone, Olanzapine, LY404039	Risperidone, Olanzapine, and LY404039 attenuated alterations in auditory-evoked potentials and gamma/alpha oscillations induced by amphetamine or PCP.
M. Fujáková et al., 2014 [142]	EEG	LY379268	LY379268 completely prevented the ketamine-induced power increase in high-frequency bands and partially improved the decrease in EEG coherence exerted by ketamine. LY379268 also reversed the hyperlocomotion due to ketamine administration.
N. C. Jones et al., 2012 [143]	EEG	Clozapine, Haloperidol, LY379268	Clozapine, Haloperidol, and LY379268 inhibited ketamine-induced hyperlocomotion, although only LY379268 prevented the ketamine-induced elevation in gamma power.
Wood et al., 2018 [144]	EEG	LY354740, LY379268	LY354740 reversed psychotic-like behaviors induced by amphetamine and PCP in control Wistar but not in Han Wistar rats. LY379268 modified the network oscillatory activity, reducing the power of high-frequency gamma bands in control Wistar but not in Han Wistar rats.
Depoortère et al., 2005 [145]	Electrophysiology	SSR504734	SSR504734 reduced the ketamine-induced increase of brain metabolic activity in the PFC, cingulate cortex, hippocampus, nucleus accumbens, and anteroventral thalamic nucleus and reversed the increase of the absolute power of the alpha1 band in rats treated with MK-801, along with a decrease in psychotic-like behaviors.
Byun et al., 2014 [146]	phMRI	VU0152100	VU0152100 antagonized amphetamine-mediated psychotic-like behaviors and modulated regional patterns of functional connectivity in the nucleus accumbens, retrosplenial cortex, motor cortex, hippocampus, and medial thalamus, together with a reversion of the amphetamine-induced elevation of extracellular dopamine levels in the nucleus accumbens and caudate-putamen.

5-HTR = serotonin receptor; BOLD = blood oxygenation level dependent; D2R = dopamine D2 receptor; EEG = electroencephalogram; fMRI = functional magnetic resonance; mVS = medial ventral striatum; PCP = phencyclidine; PFC = prefrontal cortex; phMRI = pharmacological magnetic resonance; V1 = visual cortex.

## 8. Discussion

In the present systematic review, we attempted to dissect the neurobiological basis of antipsychotics’ synaptic effects, by highlighting the close relationship between synaptic plasticity and functional connectivity and provide a comprehensive topographical analysis of antipsychotic-induced modifications in brain activity.

Alterations at the PSD level may impair the capability of neurons to communicate with each other and may account for an overall dysconnectivity between brain regions [18]. On the other hand, modifications in brain networks involved in the regulation of human behavior and cognition may directly result in psychotic symptoms [17]. Thus, the biological chain linking disruptions in synaptic plasticity with psychopathological manifestations through an impairment in functional connectivity may be explicative of the altered processes underlying schizophrenia. In this regard, functional but not structural imaging could mirror, on the macroscale, PSD alterations localized at the molecular level and macroscopic impairments in anatomical connections should be considered mainly for their consequences on brain activity [18,28].

Moreover, a global disruption in brain networks, including both increases and decreases in functional connectivity, as detected within the DMN and salience network, may underlie psychopathological manifestations in schizophrenia patients [10,11,147,148,149]. Of interest, preclinical studies showed that NMDAR antagonists, used to reproduce psychotic-like behaviors in animals, could induce both hypo- and hypersynchrony between the same cortical regions, translationally mimicking the same features that can be tracked down in the dysregulated networks of schizophrenia neurobiology [40,129,133,137]. In fact, a stable and robust re-organization of brain networks in a pathological conformation may sustain the persistency of psychotic disorders and account for resistance to canonical antipsychotics [18]. Thus, early identification and treatment of psychotic disorders could improve clinical outcomes and responsiveness to antipsychotics probably also by preventing the consolidation of altered brain connections [150,151].

A replicated feature found in the included preclinical studies was the detrimental consequence of NMDAR antagonists, such as PCP, MK-801, and ketamine, on prefrontal and hippocampal activity [40,127,145]. Given the key role in regulating high-level cognitive functions, including learning, behavioral flexibility, novelty processing, and memory, alterations in the hippocampus–PFC pathway have a remarkable translational value [127]. In this framework, dysconnectivity within networks comprising both hippocampus and PFC was detected in patients affected by schizophrenia and proposed as a possible driver of impairments in the working memory domain [152,153]. During working memory tasks, hippocampal deactivation and coordinated dorsolateral PFC enhancement are necessary to ensure the proper abolition of interfering cognitive processes [154]. In addition, ketamine has been demonstrated to disrupt striatal–PFC connectivity [134,145], which was also unveiled in patients affected by schizophrenia and psychotic bipolar disorders [155]. Taken together, these findings highlight the feasibility of NMDAR antagonism to model some aspects of psychosis’ pathophysiology, which represents the first stage in testing the efficacy of canonical and uncanonical antipsychotic agents.

Overall, antipsychotics were found to effectively antagonize the psychotic-like phenotype induced in a preclinical setting by the administration of NMDAR antagonists, along with modifications in the functional connectivity and IEGs expression profiles [128]. In an attempt to shed light on the specific pattern of molecular changes induced by individual antipsychotics, several differences between first- and second-generation compounds were outlined, as detailed in Table 1 and Table 2. Typical antipsychotics, such as haloperidol, showed to modulate especially but not exclusively striatal activity, and this modulation, at least in part, was associated with an elevation in the IEGs expression after both acute and chronic challenges [86,93,95]. Otherwise, haloperidol was demonstrated to inconsistently modulate cortical activity by both increasing and decreasing IEGs levels [79,85]. On the other hand, atypical antipsychotics, including quetiapine, olanzapine, and clozapine, were effective in regulating the cortical expression of IEGs, specifically *BDNF* in the hippocampus and PFC [103,104,105,106,107,108], as well as affecting subcortical stations, including dorsal and ventral striatum [87,88]. Clozapine, the only drug approved for treatment-resistant schizophrenia [156], was found to fine-tune cortical functional connectivity, neuronal synchrony in high-gamma bands, and PFC-medial ventral striatum coherence by reverting the alterations induced by PCP and ketamine in preclinical models via modulation of serotonin receptors [40,127,129,134]. Therefore, the beneficial action of first-generation compounds on psychotic symptoms may rely on their capability to modulate subcortical-cortical networks by tackling the dopamine receptors sited at the striatal pathways, whereas clozapine and other atypical antipsychotics may mainly target functional circuits linking the hippocampus and ventral striatum with the PFC [8,9,128,129,134]. Even novel molecules with presumptive antipsychotic efficacy, such as modulators of the NMDAR, mGluR, adrenergic, serotonergic, and muscarinic receptors, were tested in a preclinical setting and showed to be able to counteract the effects of psychomimetic compounds by affecting the structure of functional networks and cortical synchrony [127,140,145,146].

Clinical studies conducted in patients affected by schizophrenia have assessed the capability of antipsychotics to modulate differently brain activity and functional networks [157,158,159,160,161]. Specifically, monotherapy with atypical antipsychotics, such as risperidone, in drug-naive first-episode psychosis patients, showed to significantly increase functional connectivity, measured by resting-state fMRI, between the cingulate cortex and the PFC, along with improvement in positive symptoms [157]. In addition, clozapine efficacy was proven to be correlated to modifications in the functional connectivity between caudate and frontal and parietal regions, without any significant changes in striatal dopamine functioning [158]. Clozapine was also found to enhance power in the alpha, delta, and theta bands in the EEG of patients diagnosed with schizophrenia, with a more pronounced effect in frontal areas [159]. In schizophrenia patients, positron emission tomography (PET) with the application of [^15^O]-water highlighted differences between clozapine and haloperidol in the modulation of regional cerebral blood flow [160]. Clozapine was more effective in increasing cingulate cortex and PFC blood flow, whereas haloperidol had a more significant effect on striatal regions [160]. Overall, these findings confirmed the expectations inferred from preclinical studies. These results suggest tracking down functional connectivity for putative early detection in patients with psychosis to potentially help in selecting individuals who may benefit from specific drugs that can precisely target those regions involved. Future prospective clinical trials will help define predictive models of response/resistance to antipsychotics based on brain connectivity information and combined with genetic and clinical factors [162,163,164,165,166].

For the purpose of translating the present results into clinical practice terms, some limitations must be kept in mind, including the presence of a small number of papers on the topic, the high heterogeneity among studies, and the ones intrinsic to animal models. In fact, animal models cannot fully mirror the complexity of human behaviors and only indirectly reflect psychotic phenotypes, especially for cognitive dysfunction and negative symptoms of schizophrenia [167]. The pharmacological treatments modeling psychotic-like behaviors could affect different neurotransmitter pathways and represent a further source for heterogeneity in terms of antipsychotics-induced IEGs expression, brain connectivity, and effectiveness in counteracting behavioral abnormalities [168]. In this regard, most studies included in the present review evaluated changes in fMRI and electrophysiological parameters exerted by antipsychotics in murine models of psychosis, especially but not exclusively set by the administration of NMDAR antagonists. However, antipsychotics’ effects on functional connectivity have been assessed also after amphetamine administrations as well as in animals that did not model psychotic disorders, as presented in Table 2. Although the specific drug used to mirror psychosis in the preclinical setting may affect the variability of the results, it should be remembered that patients with schizophrenia also showed alterations in different brain pathways, as highlighted by PET studies [169,170,171,172,173,174,175,176,177,178]. Thus, the application of drugs capable to reproduce changes in different neurotransmitter systems, including the dopaminergic and glutamatergic ones, is relevant in terms of translatability and clinical implication, through the opportunity to explore the effects of antipsychotics on different backgrounds and by providing the basis for tailoring the therapeutic choice according to the patient-specific characteristics. For what concerns IEGs expression, most studies explored the consequences of antipsychotic administrations in animals without psychotic-like phenotypes, as shown in Table 1. In this case, the high variability detected between studies could be mostly explained by the combination of multiple factors. Among others, different drug doses, acute or chronic administrations, and genes’ specificity were the main variables involved in determining heterogeneity [78,79,85,87,179].

Since animal models never fully reflect the disease state observed in patients with psychosis, neuroimaging application in humans remains essential to study in vivo the modifications specific to the pathology and those exerted by antipsychotics. Thus, preclinical and clinical models may provide complementary information, which is indispensable to explore psychotic disorders from different perspectives.

Further studies will be needed to precisely define the different effects of each antipsychotic to modulate interregional activity in the animal and human brain, grouping results according to different drug doses, genes considered, and administration paradigms. The application of novel techniques, such as HCR RNA-ISH, will be a further step toward the understanding of the molecular changes induced by antipsychotics and their potential impact on psychopathological manifestations, due to the unique opportunity to associate topographic and transcriptomic information [120].

Despite the limitations, the preclinical setting may offer a fertile background for testing the possible effectiveness of novel compounds with putative antipsychotic activity by directly targeting the neurobiological core of psychosis, such as alterations in synaptic plasticity and brain functional connectivity. The preclinical investigation could be instrumental to overcome some limitations of human studies, including confounding variables, the issue of undirect measures of brain activity, and the low-spatial resolution resulting from fMRI and EEG applications. The opportunity to explore the specific pattern of molecular changes induced by different compounds characterized by different pharmacodynamic profiles represents a prerequisite for creating predictive models of drug response or resistance. Lastly, preclinical studies could provide translational findings for the development of more focused therapeutic strategies, for instance, by matching information on the topography of brain activity induced by different antipsychotics with patient-specific alterations in functional connectivity profiles.

## Data Availability

Not applicable.

## Supplementary Materials

The following supporting information can be downloaded at: https://www.mdpi.com/article/10.3390/biomedicines10123183/s1, the search strings are provided in the Supplementary Materials.
