# Supplementary material for: Antipsychotics-Induced Changes in Synaptic Architecture and Functional Connectivity: Translational Implications for Treatment Response and Resistance
Source: Biomedicines. 2022 Dec 8;10(12):3183. doi: 10.3390/biomedicines10123183 (PMC9776416; doi:10.3390/biomedicines10123183)
Supplement: Supplementary file 1 [file biomedicines-10-03183-s001.zip › biomedicines-2027105-supplementary.pdf]

Supplementary material

Search string on Pubmed: ("arc"[Title/Abstract] OR "arg"[Title/Abstract] OR "bdnf"[Title/Abstract] OR "fos"[Title/Abstract] OR "c fos"[Title/Abstract] OR "homer"[Title/Abstract] OR "jun"[Title/Abstract] OR "c jun"[Title/Abstract] OR "egr1"[Title/Abstract] OR "zif268"[Title/Abstract] OR "narp"[Title/Abstract] OR "npas"[Title/Abstract] OR "nor1"[Title/Abstract] OR "nurr"[Title/Abstract] OR "nerve growth factor inducible"[Title/Abstract] OR "ish"[Title/Abstract] OR "in situ hybridization"[Title/Abstract] OR "fmri"[Title/Abstract] OR "eeg"[Title/Abstract] OR "network"[Title/Abstract] OR "connectivity"[Title/Abstract] OR "connectome"[Title/Abstract]) AND ("antipsychotic"[Title/Abstract] OR "chlorpromazine"[Title/Abstract] OR "haloperidol"[Title/Abstract] OR "paliperidone"[Title/Abstract] OR "risperidone"[Title/Abstract] OR "asenapine"[Title/Abstract] OR "olanzapine"[Title/Abstract] OR "clozapine"[Title/Abstract] OR "quetiapine"[Title/Abstract] OR "amisulpride"[Title/Abstract] OR "aripiprazole"[Title/Abstract] OR "brexpiprazole"[Title/Abstract] OR "cariprazine"[Title/Abstract] OR "lurasidone"[Title/Abstract])

Search string on Embase: ('arc':ab,ti OR 'bdnf':ab,ti OR 'c fos':ab,ti OR 'homer1':ab,ti OR 'c jun':ab,ti OR 'egr1':ab,ti OR 'zif268':ab,ti OR 'narp':ab,ti OR 'npas':ab,ti OR 'nor1':ab,ti OR 'nurr':ab,ti OR 'nerve growth factor inducible':ab,ti OR 'network':ab,ti OR 'connectivity':ab,ti OR 'connectome':ab,ti) AND ('antipsychotic':ab,ti OR 'chlorpromazine':ab,ti OR 'haloperidol':ab,ti OR 'paliperidone':ab,ti OR 'risperidone':ab,ti OR 'asenapine':ab,ti OR 'olanzapine':ab,ti OR 'clozapine':ab,ti OR 'quetiapine':ab,ti OR 'amisulpride':ab,ti OR 'aripiprazole':ab,ti OR 'brexpiprazole':ab,ti OR 'cariprazine':ab,ti OR 'lurasidone':ab,ti)
